# Safety of reduced antibiotic prescribing for self limiting respiratory tract infections in primary care: cohort study using electronic health records

**DOI:** 10.1136/bmj.i3410

**Published:** 2016-07-05

**Authors:** Martin C Gulliford, Michael V Moore, Paul Little, Alastair D Hay, Robin Fox, A Toby Prevost, Dorota Juszczyk, Judith Charlton, Mark Ashworth

**Affiliations:** 1 Department of Primary Care and Public Health Sciences, King’s College London, Guy’s Campus, London SE1 1UL, UK; 2 Academic Unit for Primary Care and Population Sciences, University of Southampton, Southampton, UK; 3 Centre for Academic Primary Care, School of Social and Community Medicine, University of Bristol, Bristol, UK; 4 The Health Centre, Bicester, Oxford, UK

## Abstract

**Objective** To determine whether the incidence of pneumonia, peritonsillar abscess, mastoiditis, empyema, meningitis, intracranial abscess, and Lemierre’s syndrome is higher in general practices that prescribe fewer antibiotics for self limiting respiratory tract infections (RTIs).

**Design** Cohort study.

**Setting** 610 UK general practices from the UK Clinical Practice Research Datalink.

**Participants** Registered patients with 45.5 million person years of follow-up from 2005 to 2014.

**Exposures** Standardised proportion of RTI consultations with antibiotics prescribed for each general practice, and rate of antibiotic prescriptions for RTIs per 1000 registered patients.

**Main outcome measures** Incidence of pneumonia, peritonsillar abscess, mastoiditis, empyema, meningitis, intracranial abscess, and Lemierre’s syndrome, adjusting for age group, sex, region, deprivation fifth, RTI consultation rate, and general practice.

**Results** From 2005 to 2014 the proportion of RTI consultations with antibiotics prescribed decreased from 53.9% to 50.5% in men and from 54.5% to 51.5% in women. From 2005 to 2014, new episodes of meningitis, mastoiditis, and peritonsillar abscess decreased annually by 5.3%, 4.6%, and 1.0%, respectively, whereas new episodes of pneumonia increased by 0.4%. Age and sex standardised incidences for pneumonia and peritonsillar abscess were higher for practices in the lowest fourth of antibiotic prescribing compared with the highest fourth. The adjusted relative risk increases for a 10% reduction in antibiotic prescribing were 12.8% (95% confidence interval 7.8% to 17.5%, P<0.001) for pneumonia and 9.9% (5.6% to 14.0%, P<0.001) for peritonsillar abscess. If a general practice with an average list size of 7000 patients reduces the proportion of RTI consultations with antibiotics prescribed by 10%, then it might observe 1.1 (95% confidence interval 0.6 to 1.5) more cases of pneumonia each year and 0.9 (0.5 to 1.3) more cases of peritonsillar abscess each decade. Mastoiditis, empyema, meningitis, intracranial abscess, and Lemierre’s syndrome were similar in frequency at low prescribing and high prescribing practices.

**Conclusions** General practices that adopt a policy to reduce antibiotic prescribing for RTIs might expect a slight increase in the incidence of treatable pneumonia and peritonsillar abscess. No increase is likely in mastoiditis, empyema, bacterial meningitis, intracranial abscess, or Lemierre’s syndrome. Even a substantial reduction in antibiotic prescribing was predicted to be associated with only a small increase in numbers of cases observed overall, but caution might be required in subgroups at higher risk of pneumonia.

## Introduction

Concern is growing that the widespread and sometimes unnecessary use of antibiotics is leading to the development of antimicrobial drug resistance and potentially to infections caused by resistant organisms that are difficult to treat.^1^ Reducing the inappropriate use of antibiotics, as well as ensuring that they can be used when needed, represent important components of a strategy to control infectious diseases.^2^ In the healthcare sector, attention has focused on primary care, including first point-of-contact ambulatory care, where a high proportion of antibiotics are prescribed. About 60% of antibiotics prescribed in primary care are for respiratory tract infections (RTIs).^3^ RTIs, including common colds, sore throat, cough, acute bronchitis, otitis media, and sinusitis are often self limiting and usually improve without specific treatment.^4^ Antibiotic treatment of RTIs offers negligible benefit to affected patients^5^ and is often associated with side effects.^6^ Guidance in the United Kingdom recommends that either a no antibiotic prescribing strategy or a delayed antibiotic prescribing strategy should be agreed for most patients with RTIs.^3^ Nevertheless, around 36% of common colds continue to be treated with antibiotics, as do 40% of episodes of sore throat, 70% of otitis media, and 90% of sinusitis.^7^ Wide variation exists among general practices. About 50% of all general practice consultations for RTIs result in an antibiotic prescription, but some general practices issue prescriptions at a rate of more than 80% and others at less than 20%.^8^ This contrasts with practice in the Netherlands, where 22.5% of RTI episodes in 2010 were treated with antibiotics.^9^ A considerable amount of research has been done^10^ ^11^ ^12^ to develop interventions that might help general practitioners to reduce the rate of antibiotic prescribing for RTIs. This has been translated into policy guidance^3^ and public campaigns^13^ to control unnecessary antibiotic use.

Clinical concern that reducing antibiotic use might increase the risk of complications following RTIs might be realistic. Evidence from clinical trials suggests that antibiotics may reduce the risk of suppurative complications of RTIs,^5^ but the more serious complications are generally too rare to evaluate precisely in randomised studies. A cohort study in 162 general practices in the General Practice Research Database from 1991 to 2001 evaluated the effect of antibiotic treatment on the incidence of pneumonia after upper RTI, peritonsillar abscess after sore throat, and mastoiditis after otitis media.^14^ The results suggested that antibiotic treatment was associated with lower odds of each of these complications, but the overall risk of complications was generally small and the number of patients who would have to be treated to avoid one complication was estimated to be in excess of 4000. However, pneumonia was more common; in people aged 65 or more an estimated one case for every 39 antibiotic prescriptions might be avoided. Infections of the middle ear or sinuses may rarely be complicated by intracranial abscess.^15^ Lemierre’s syndrome,^16^ from thrombophlebitis of the internal jugular vein associated with *Fusobacterium necrophorum* infection, is a rare complication of sore throat,^16^ but *F necrophorum* might be frequently detectable in patients with symptoms of sore throat.^17^ ^18^ The annual number of cases of Lemierre’s syndrome in England was reported to have increased from 19 in 1997 to 34 in 1999, prompting a reminder from the chief medical officer that some symptoms of sore throat may require antibiotic treatment.^19^ In addition to concerns about complications, medical practitioners may be concerned about the potential consequences of diagnostic misclassification. The initial symptoms of meningitis may sometimes resemble an influenza-like illness.^20^ Awareness of the possibility of a more serious diagnosis might prompt general practitioners to issue an antibiotic prescription for conditions in which antibiotics are not usually indicated.

These observations raise important questions for a policy to reduce antibiotic prescribing for RTIs in primary care: Is there a safe level of antibiotic prescribing for RTIs? What target can general practices safely adopt in reducing the proportion of consultations for RTIs with antibiotics prescribed? Is there a threshold for antibiotic prescribing below which complications may increase? We evaluated the safety of a policy to reduce antibiotic prescribing for RTIs in primary care and the incidence of pneumonia, peritonsillar abscess, mastoiditis, empyema, meningitis, intracranial abscess, and Lemierre’s syndrome. We determined whether these complications were more common in general practices that prescribe fewer antibiotics for self limiting RTIs than higher prescribing practices. We aimed to use this information to quantify the potential clinical and public health impact of changes in antibiotic prescribing practice.

## Methods

The data source for the study was the UK Clinical Practice Research Datalink (CPRD).^21^ This is a large database containing fully anonymised electronic records from about 7% of UK general practices from 1987 to the present. CPRD data are considered representative of the UK population, and the high quality of CPRD data have been confirmed in many studies.^21^ For the present study, we included data for the 10 year period from 2005 to 2014. During this period the CPRD included data for an open cohort of about 4.5 million registered patients.

### Definition of infective complications of RTIs

We evaluated the number of first episodes of infective complications in the entire registered population of CPRD from 2005 to 2014. Such complications were defined using Read medical codes recorded in participants’ electronic health records. The Read code classification represents a terminology used to code primary care electronic health records in the UK.^22^ Electronic health records include diagnoses recorded at primary care consultations and home visits. In addition, the CPRD referral file includes coded data for hospital referrals and hospital discharges. In analyses we evaluated pneumonia (57 codes), empyema (14 codes), peritonsillar abscess (5 codes), mastoiditis (13 codes), bacterial meningitis (19 codes), and intracranial abscess (14 codes). Codes for “pneumonia” were drawn from section H2 of the Read code classification, which includes codes for “pneumonia and influenza.” Codes were included if they indicated the presence of pneumonia without a viral cause. “Bacterial meningitis” included codes for meningococcal meningitis, meningococcal septicaemia, pneumococcal meningitis, and haemophilus meningitis, as well as unspecified bacterial meningitis. Code lists are available from the authors. Data were extracted for all participants with records of infective complications from 2005 to 2014. We defined incident events as the first record of an event in a participant that was recorded more than 12 months after the start of the participant’s CPRD record. Sex, year, and age group were included as individual level covariates. Nine 10 year age groups were employed, with categories of 0 to 14 years and 85 years or more. We aggregated incident events by year, age group, sex, and general practice. Person time for the registered CPRD population was estimated by year, age group, sex, and general practice to estimate rates of infective complications. Cluster level covariates included CPRD region, with 10 regions in England, as well as Wales, Scotland, and Northern Ireland. Deprivation fifth was included, based on general practice level data for indices of multiple deprivation score (IMD 2010) for England, and equivalent scores in Scotland, Wales, and Northern Ireland.

### Definition of RTI consultation and antibiotic prescribing rates

We estimated age standardised measures for RTI consultations and antibiotic prescribing as reported previously.^7^ ^23^ For each CPRD general practice we estimated the rate of RTI consultations per 1000 registered patients, the antibiotic prescribing rate for RTI per 1000 registered patients, and the proportion (%) of RTI consultations with antibiotics prescribed. These prescribing measures were estimated on a sample of CPRD data because it was not feasible and our licence did not allow us to perform the analysis on the entire CPRD database. Participants were sampled from all acceptable patients included in CPRD. A random sample of 75 currently registered patients was drawn without replacement for each year from 2005 to 2014. This gave a maximum sample of 750 participants, with up to 7500 person years of observation for each practice. We aimed to achieve a total sample of fewer than 0.5 million, and the total sample for analysis was 411 226 participants from 643 general practices. This allowed us to estimate practice specific proportions with a 1% margin of error. For participants in the sample, we estimated person years as denominator from the start of CPRD registration or 1 January 2005, to the end of the participant’s CPRD record or 31 December 2014. We identified self limiting RTIs using medical codes recorded during general practice consultations. These were classified into five groups following the recommendations of the National Institute for Health and Care Excellence^3^: colds and “upper respiratory tract infections”; sore throat, including pharyngitis and laryngitis; cough and acute bronchitis; otitis media; and rhinosinusitis. Acute bronchitis was included because current recommendations are to avoid antibiotic treatment.^3^ Consultations for RTIs were identified, and we selected first consultations within a 14 day time window. Data for participants aged 100 or older were excluded. We identified antibiotic prescriptions issued on the same day as consultations for respiratory problems and then estimated for each general practice the rate of consultations for RTIs per 1000 person years, rate of antibiotic prescribing for RTIs per 1000 person years, and proportion (%) of RTI consultations with antibiotics prescribed. Rates and proportions were standardised for age and sex using the 2013 European standard population. After excluding practices with insufficient data, because of short periods of contributing to CPRD, we estimated rates for 610 CPRD general practices.

### Statistical analysis

In the final stage of the analysis, we estimated the numbers of infective complications with person years at risk, in relation to general practice specific rates of RTI consultations and antibiotic prescribing. Mixed effects Poisson models were fitted using the hglm package^24^ in the R program.^25^ General practice was fitted as a random effect. The log of person years was included as offset. Fixed effects included sex, year, age group, region, and deprivation fifth. We evaluated the association of age standardised RTI consultation rate with rates of infective complications. After adjusting for the RTI consultation rate, we evaluated the association of the antibiotic prescribing rate and the antibiotic prescribing proportion with infective complication rates. Incident rate ratios (95% confidence intervals) were estimated for each fourth of RTI consultation rate, antibiotic prescribing rate, or antibiotic prescribing proportion, using the lowest fourth for reference. The RTI consultation rate, antibiotic prescribing rate, and antibiotic prescribing proportion were also fitted as continuous predictors, and we estimated incident rate ratios for each 10 unit change in the predictor. We evaluated whether the addition of quadratic terms improved goodness of fit. As there were small numbers of events for intracranial abscess, and mixed effects models did not converge, we omitted the random effect for general practice for this outcome. Regression models were not fitted for Lemierre’s syndrome because this condition was rare. The ggplot2^26^ and forestplot^27^ packages in R were used to present the results.

To present the clinical implications of these findings, we calculated the number of events expected in a general practice with 7000 patients (the general practice mean list size for England) during 10 years of follow-up. To estimate the expected number of consultations for RTIs we used the median (95% range) for the RTI consultation rate. To estimate expected numbers of complications and antibiotic prescriptions we used the disease incidence and distribution of antibiotic prescribing proportion for the highest prescribing fourth. We used the relative risk increase for a 10% change in antibiotic prescribing from the Poisson model to estimate the expected change in number of infective complications.

### Patient involvement

No patients were involved in setting the research question or the outcome measures, nor were they involved in developing plans for design or implementation of the study. No patients were asked to advise on interpretation or writing up of results. Results will be disseminated to relevant patient communities through news media.

## Results

Data were analysed for 610 UK Clinical Practice Research Datalink (CPRD) general practices, with 45 465 201 registered person years of observation from 2005 to 2014. Figure 1 presents data for RTI consultations and associated antibiotic prescribing between 2005 and 2014. The RTI consultation rate per 100 000 continued a long term decline^7^ during the period, decreasing from 256 to 220 per 100 000 in men and from 351 to 307 per 100 000 in women. The antibiotic prescribing rate for RTIs declined from 128 to 106 per 100 000 in men and from 184 to 155 per 100 000 in women. The proportion of RTI consultations with antibiotics prescribed declined from 53.9% to 50.5% in men and from 54.5% to 51.5% in women.

**Figure f1:**
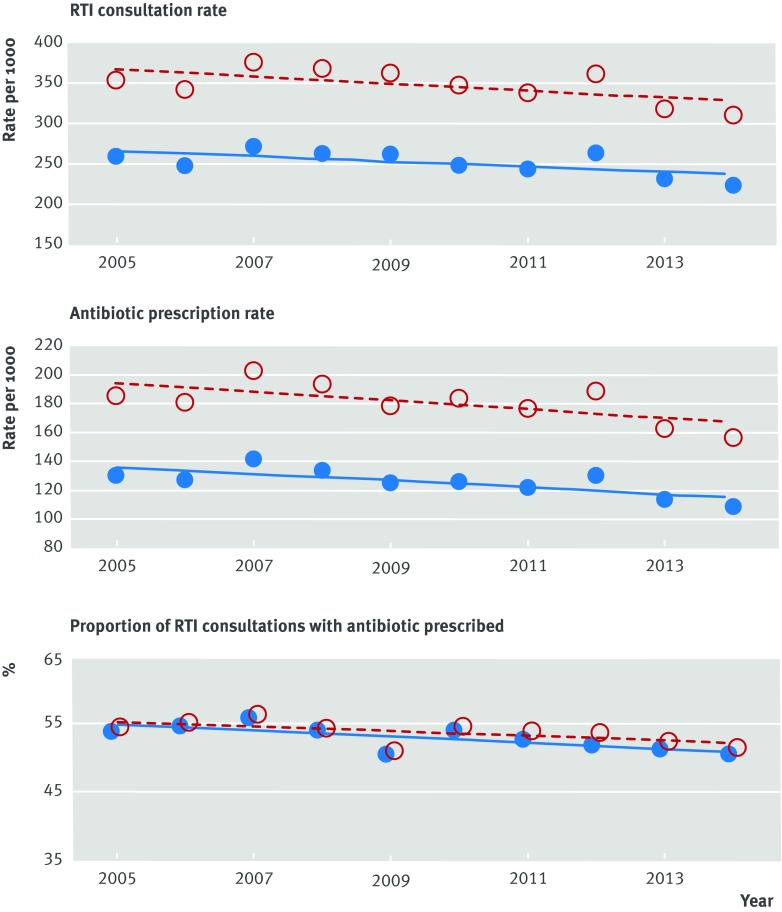
**Fig 1** Age standardised consultation rate for self limiting respiratory tract infections (RTIs), antibiotic prescribing rate for RTIs, and proportion of RTI consultations with antibiotics prescribed in 610 general practices contributing to the UK Clinical Practice Research Datalink. Red open circles represent females; blue filled circles represent males. Lines fitted by least squares

Figure 2 and table 1 show changes between 2005 and 2014 in rates of outcome measures for males and females registered in CPRD. Over the period there were declining trends in incidence of peritonsillar abscess (1% yearly), mastoiditis (4.6%), and meningitis (5.3%), pneumonia showed an increase of 0.4% yearly, and empyema and intracranial abscess showed no clear change over time.

**Figure f2:**
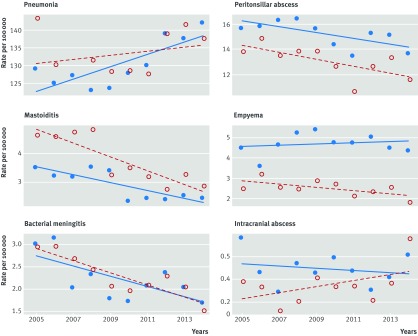
**Fig 2** Incidence of infective complications in 610 general practices contributing to the UK Clinical Practice Research Datalink. Red open circles represent females; blue filled circles represent males. Lines fitted by least squares

**Table 1 tbl1:** Annual percentage relative change in incidence of infective complications from 2005 to 2014

Infective complications	Annual % change in relative incidence (95% CI)*	P value
Pneumonia	0.36 (0.09 to 0.64)	0.008
Peritonsillar abscess	−0.99 (−1.45 to −0.53)	<0.001
Mastoiditis	−4.64 (−5.21 to −4.07)	<0.001
Empyema	−0.56 (−1.16 to 0.05)	0.07
Bacterial meningitis	−5.28 (−4.69 to 5.87)	<0.001
Intracranial abscess	−1.36 (−6.66 to 3.68)	0.60

*Adjusted for age group, sex, region, deprivation fifth, consultation rate for respiratory tract infections (RTIs), and proportion of RTI consultations with antibiotics prescribed and clustering by general practice.

General practices were divided into fourths according to the proportion of RTI consultations with antibiotics prescribed (table 2). General practices in the highest fourth prescribed antibiotics at a median 65% (range 58% to 79%) of RTI consultations, whereas general practices in the lowest fourth prescribed antibiotics at a median 38% (29% to 44%) of RTI consultations. Table 2 shows the age standardised incidence rates for each of the infective complications. The incidence of pneumonia was 157 (95% confidence interval 154 to 159) per 100 000 at low prescribing practices but 119 (117 to 121) per 100 000 at high prescribing practices. The corresponding values for peritonsillar abscess were 15.6 (15.5 to 15.8) per 100 000 and 12.9 (12.8 to 13.0) per 100 000. Mastoiditis, empyema, bacterial meningitis, and intracranial abscess showed lower incidence rates, which did not appear to be associated with antibiotic prescribing category. Fourteen cases of Lemierre’s syndrome occurred, which were evenly distributed between prescribing categories, with an overall incidence rate of 0.31 per million.

**Table 2 tbl2:** Distribution of general practices and person years follow-up for registered patients from 2005 to 2014 for 610 general practices contributing to the UK Clinical Practice Research Datalink

Variables	Fourths of proportion of RTI consultations with antibiotics prescribed			
	High ≥58%	51-57%	44-50%	Low <44%
No of general practices	152	153	152	153
No of person years from registered patients	10 573 885	12 135 183	12 109 005	10 647 128
Median (95% range) proportion of RTI consultations with antibiotics prescribed	65 (58-79)	54 (51-57)	48 (45-50)	38 (29-44)
Infective complications*:				
Pneumonia	119.2 (117.0 to 121.3)	129.1 (126.9 to 131.2)	156.4 (154.0 to 158.7)	156.6 (154.0 to 159.1)
Peritonsillar abscess	12.9 (12.8 to 13.0)	13.2 (13.1 to 13.3)	14.1 (13.9 to 14.2)	15.6 (15.5 to 15.8)
Mastoiditis	3.48 (3.37 to 3.60)	3.31 (3.21 to 3.42)	3.32 (3.19 to 3.46)	3.38 (3.25 to 3.51)
Empyema	3.64 (3.27 to 4.01)	4.00 (3.63 to 4.37)	3.66 (3.31 to 4.01)	4.00 (3.61 to 4.40)
Bacterial meningitis	2.19 (1.90 to 2.47)	2.16 (1.90 to 2.42)	2.24 (1.97 to 2.51)	2.45 (2.15 to 2.75)
Intracranial abscess	0.37 (0.25 to 0.48)	0.35 (0.24 to 0.46)	0.55 (0.42 to 0.69)	0.42 (0.29 to 0.55)
Lemierre’s syndrome	4 cases	3 cases	2 cases	5 cases

RTI=respiratory tract infection.

*Values are age and sex standardised incidence rate per 100 000 (95% CI).

Figure 3 presents the occurrence of infective complications according to the rate of RTI consultations. General practices in the highest fourth for RTI consultation rate had higher incidence rates for pneumonia and mastoiditis (1.35, 1.14 to 1.61, P=0.001 and 1.67, 1.20 to 2.33, P=0.002, respectively) compared with general practices in the lowest fourth. Peritonsillar abscess, empyema, bacterial meninigitis, and intracranial abscess were not associated with the general practice RTI consultation rate.

**Figure f3:**
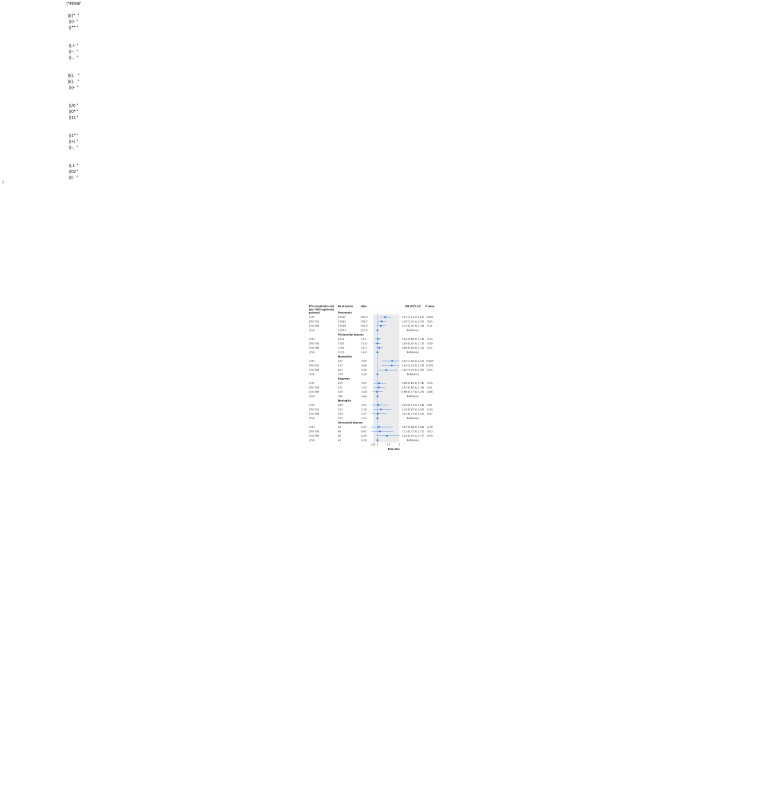
**Fig 3** Association of incidence of infective complications with fourth of consultation rate for self limiting respiratory tract infections (RTIs). Rates are number of incident events per 100 000 person years. Incidence rate ratios (IRRs) were adjusted for sex, age group, region, deprivation fifth, and clustering by general practice

Figure 4 shows the association between the proportion of RTI consultations with antibiotics prescribed and the incidence of infective complications. The risk of pneumonia and peritonsillar abscess decreased as the antibiotic prescribing proportion for RTI increased, but there was no clear evidence of an association for mastoiditis, empyema, meningitis, and intracranial abscess. For general practices in the highest fourth of antibiotic prescribing, the incidence rate ratio for pneumonia was 0.70 (95% confidence interval 0.59 to 0.82, P<0.001), and for peritonsillar abscess was 0.78 (0.68 to 0.90, P<0.001) compared with the lowest fourth.

**Figure f4:**
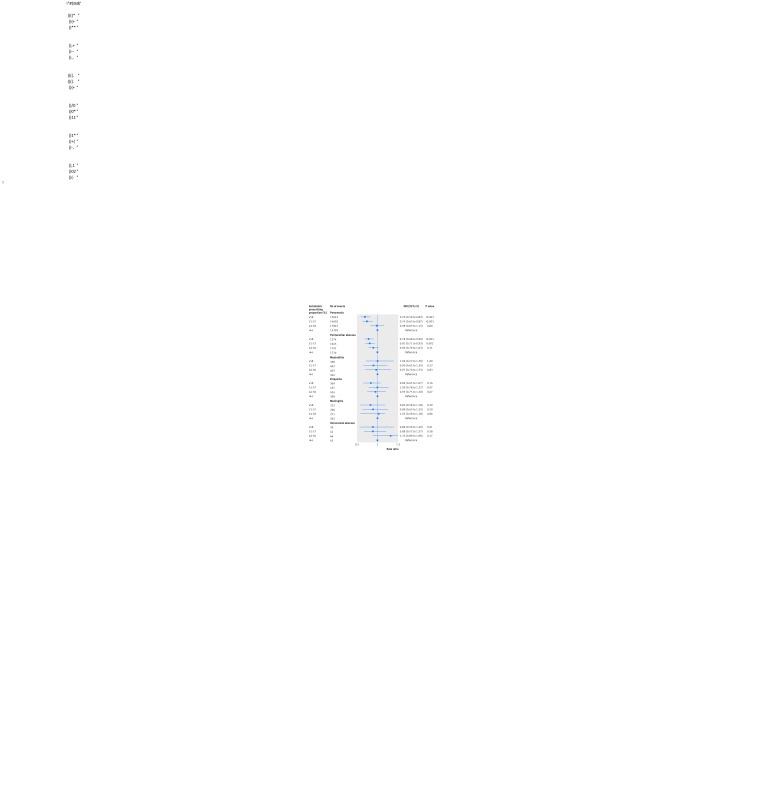
**Fig 4** Association of incidence of infective complications with fourth of antibiotic prescribing proportion. Incidence rate ratios (IRRs) were adjusted for consultation rate for respiratory tract infections, sex, age group, region, deprivation fifth, and clustering by general practice

Figure 5 shows the association between the antibiotic prescribing rate and infective complications. Pneumonia showed an association with the antibiotic prescribing rate (incidence rate ratio for highest fourth 0.74, 0.58 to 0.95, P=0.02). Peritonsillar abscess showed a weak association (0.84, 0.68 to 1.03, P=0.09) but the other infective complications did not. The antibiotic prescribing rate is determined by the RTI consultation rate and the proportion of consultations with antibiotics prescribed. It is correlated with both the RTI consultation rate (r=0.82) and the antibiotic prescribing proportion (r=0.66).

**Figure f5:**
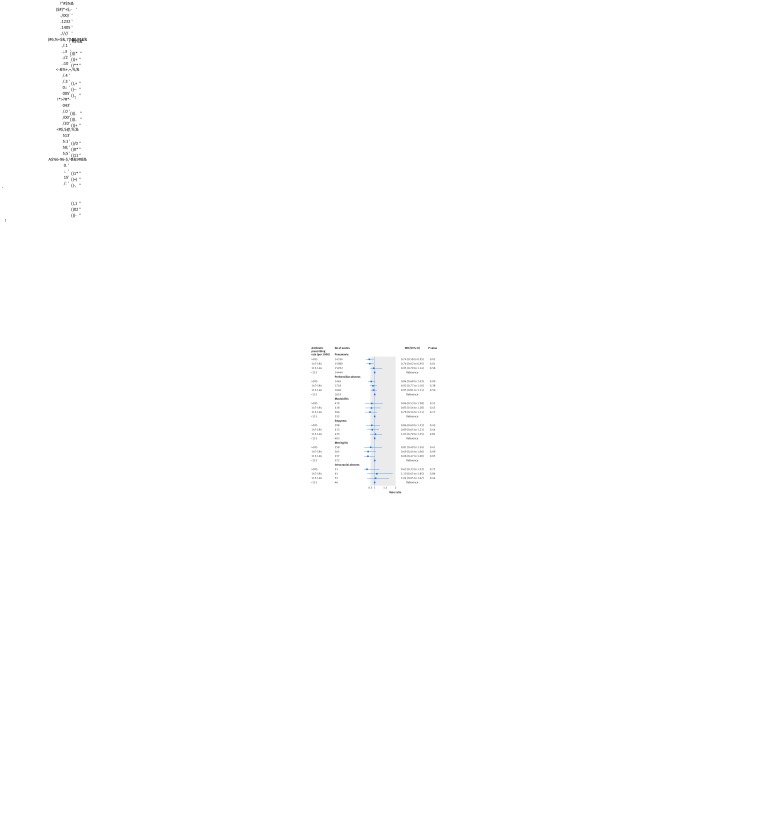
**Fig 5** Association of incidence of infective complications with fourth of antibiotic prescribing rate. Incidence rate ratios (IRRs) were adjusted for consultation rate for respiratory tract infections, sex, age group, region, deprivation fifth, and clustering by general practice

Table 3 shows incident rate ratios estimated after fitting the predictors as continuous variables. These estimates are consistent with linear associations; adding quadratic terms did not improve the goodness of fit. An increasing RTI consultation rate was associated with an increasing incidence of pneumonia and mastoiditis. An increasing antibiotic prescribing proportion was associated with a declining incidence of pneumonia and peritonsillar abscess. Each 10% increase in antibiotic prescribing proportion was associated with a 12.8% (95% confidence interval 7.8% to 17.5%) relative decrease in pneumonia and a 9.9% (5.6% to 14.0%) decrease in peritonsillar abscess. Associations with the antibiotic prescribing rate fitted as a linear predictor were consistent with those for the antibiotic prescribing proportion. In additional analyses, we found that the incidence rate ratio associating the antibiotic prescribing proportion with pneumonia was similar for the population aged less than 65 years and 65 years or older.

**Table 3 tbl3:** Associations of consultation and prescribing rates and proportions with infective complications. Incident rate ratios (IRR) are for a 10 unit increment in rate or proportion

Infective complications	RTI consultation rate (for 10 per 1000 increase)			Antibiotic prescribing rate for RTI (for 10 per 1000 increase)			Proportion of RTI consultations with antibiotic prescribed (for 10% increase)	
	IRR* (95% CI)	P value		IRR† (95% CI)	P value		IRR† (95% CI)	P value
Pneumonia	1.015 (1.008 to 1.023)	<0.001		0.959 (0.941 to 0.976)	<0.001		0.87 (0.83 to 0.92)	<0.001
Peritonsillar abcess	1.004 (0.998 to 1.010)	0.18		0.968 (0.953 to 0.982)	<0.001		0.90 (0.86 to 0.94)	<0.001
Mastoiditis	1.020 (1.005 to 1.034)	0.007		1.008 (0.973 to 1.044)	0.67		1.00 (0.90 to 1.12)	0.95
Empyema	1.005 (0.995 to 1.016)	0.35		0.979 (0.953 to 1.005)	0.11		0.93 (0.86 to 1.01)	0.10
Bacterial meninigitis	1.001 (0.987 to 1.016)	0.86		0.986 (0.949 to 1.023)	0.45		0.94 (0.84 to 1.06)	0.30
Intracranial abscess‡	1.003 (0.984 to 1.022)	0.75		0.986 (0.938 to 1.035)	0.57		0.94 (0.81 to 1.09)	0.40

*Adjusted for sex, age group, region, deprivation fifth, and clustering by general practice.

†Adjusted for consultation rate for respiratory tract infections, sex, age group, region, deprivation fifth, and clustering by general practice.

‡Adjustment for general practice omitted.

A general practice with the mean list size for England of 7000 registered patients is expected to have 20 300 consultations (95% range 11 340 to 30 380) for RTIs over 10 years (table 4). A general practice of this size, with an average RTI consultation rate, might issue 13 195 (11 744 to 16 037) antibiotic prescriptions during this period if it is in the highest prescribing fourth. If the practice reduces the proportion of RTI consultations with antibiotics prescribed by 10% it will issue 2030 (1134 to 3038) fewer antibiotic prescriptions for RTIs. This reduction in antibiotic prescribing is expected to be associated with 1.1 (0.6 to 1.5) more cases of pneumonia each year and 0.9 (0.5 to 1.3) more cases of peritonsillar abscess each decade (table 4). The number of cases of mastoiditis, empyema, bacterial meningitis, intracranial abscess, and Lemierre’s syndrome are not expected to increase.

**Table 4 tbl4:** Expected number of events over 10 years in a hypothetical high antibiotic prescribing general practice with 7000 patients

Measures	Median (95% range) over 10 years	
	No expected in general practice with 7000 patients	Change after 10% absolute decrease in proportion of RTI consultations with antibiotics prescribed
No of RTI consultations	20 300 (11 340 to 30 380)	0
Antibiotic prescriptions for RTI	13 195 (11 744 to 16 037)*	−2030 (−1134 to −3038)†
No of first episodes:		
Pneumonia	83 (82 to 85)	11 (6 to 15)
Peritonsillar abscess	9 (9 to 9)	0.9 (0.5 to 1.3)
Mastoiditis	2 (2 to 3)	0
Empyema	3 (2 to 3)	0
Bacterial meningitis	2 (1 to 2)	0
Intracranial abscess	<1	0

RTI=respiratory tract infection.

*Assuming average RTI consultation rate.

†Assuming no change in RTI consultation rate.

## Discussion

We used a large dataset of electronic health records to investigate the safety of reducing unnecessary antibiotic prescribing for respiratory tract infections (RTIs) in primary care. The results show that general practices prescribing fewer antibiotics for RTIs may expect to have a slightly higher incidence of pneumonia and peritonsillar abscess than higher prescribing general practices. If a general practice with an average list size of 7000 patients reduced the proportion of RTI consultations with antibiotics prescribed by 10%, it might encounter about one additional case of pneumonia each year and one additional case of peritonsillar abscess each decade. Changes will be proportionately greater for larger reductions in antibiotic prescribing. These estimates represent averages across general practice populations, but complications might be fewer than expected if general practitioners are able effectively to stratify antibiotic prescribing according to level of risk. There was no evidence that diagnoses of mastoiditis, empyema, bacterial meningitis, or intracranial abscess might increase. Lemierre’s syndrome was rare, with about one case per two million person years, but there was no evidence that this was more common at low prescribing practices. This is reassuring in view of recent suggestions that *F necrophorum* may often be present in patients with sore throat.^18^ These estimates must be viewed in the context of quantitatively important declining secular trends in incidence for several infective complications of RTI, including peritonsillar abscess, mastoiditis, and meningitis. Bacterial meningitis from pneumococcal, meningococcal, or haemophilus infection has declined after the introduction of vaccination programmes.^28^ However, the incidence of pneumonia showed a slight increase over time, consistent with previous studies based on hospital admissions.^29^ ^30^

Reducing the proportion of RTI consultations with antibiotics prescribed by 10% is expected to be accompanied by some 2000 fewer antibiotic prescriptions for each practice over 10 years. Benefits to individual patients from avoiding antibiotics include reductions in common adverse reactions to antibiotics, such as rashes, vomiting, and diarrhoea, which may affect 10% of patients,^6^ as well as less common side effects such as anaphylaxis. Benefits to general practices may include a demedicalisation of RTIs followed by a decline in the rate of consultations, since previous observational studies show that higher prescribing general practices receive more consultations for RTIs.^31^ Trial evidence shows that even one antibiotic prescription increases the likelihood of reconsultation for a new episode of an RTI.^32^ Most of the complications identified do not require hospital admission and currently respond well to antibiotics, so simply the occurrence of an uncommon complication rate is not in itself a justification for more widespread prescribing of antibiotics for initially uncomplicated presentations. The results did not support a threshold for safe or unsafe prescribing levels. Inspection of forest plots suggested some departure from linearity, but addition of non-linear terms did not improve the goodness of fit of regression models.

### Strengths and weaknesses in relation to other studies

Previous studies have consistently shown high levels of unnecessary prescribing of antibiotics for RTIs in primary care.^33^ While there has been a declining trend in the consultation rate for RTIs,^7^ the proportion of consultations with antibiotics prescribed has changed little,^7^ despite the efforts of researchers, clinicians, and policymakers to bring about changes. General practitioners may often be concerned to meet patients’ expectations for antibiotic prescriptions,^34^ but both patients and prescribers might also have concerns about the safety of non-prescribing strategies.^34^ One study^14^ provided evidence that antibiotics reduced the risk of pneumonia, mastoiditis, and peritonsillar abscess but did not quantify the potential population impact of these complications.

The present results represent averages across general practice populations. Diversity among the population of patients at risk of RTIs is considerable. Current management guidelines for RTIs recommend that specific groups of patients should be considered to have positive indications for antibiotic treatment. An immediate antibiotic prescription is recommended if patients have clinical features suggestive of serious illness or complications,^35^ have comorbidities, or are very young or very old.^3^ Further research is needed to evaluate whether the present results will be confirmed when subgroups that might be at higher risk, including older adults, are analysed separately. It is possible that general practices with the same overall level of antibiotic prescribing may differ in the appropriateness of their management of patients with defined markers of vulnerability, and this could influence the rate of complications. However, the clinical features of an RTI episode may have only limited predictive value for the future occurrence of complications, and a high proportion of complications might occur in patients who seem to be at low risk.^36^ A delayed antibiotic prescribing strategy, in which a prescription is issued but only used if symptoms fail to improve, is sometimes recommended as a method for reducing antibiotic utilisation in the management of RTIs.^3^ ^37^ Delayed antibiotic prescribing may be as effective as immediate use of antibiotics in the prevention of complications of sore throat.^4^ The development and application of point-of-care testing to guide antibiotic prescribing might have a future role in identifying those who would potentially benefit from antibiotic treatment.^38^ ^39^

### Strengths and weaknesses of this study

This study included more than 600 general practices, with a registered population of more than four million patients and 45 million person years of observation. Consequently, the study provided precise estimates for the more common outcomes evaluated. We acknowledge that there was lower power to evaluate potential changes in less common outcomes. We can conclude that the absolute risks of mastoiditis, empyema, intracranial abscess, and Lemierre’s syndrome remain small, even in practices with low rates of antibiotic prescribing. This study adopted a population perspective, aiming to quantify the outcomes of either high prescribing or low prescribing strategies in the management of RTIs. Consequently, we evaluated changes in infective complications at the level of the general practice population. The research did not deal with variation in prescribing at the level of the individual doctor. The research did not show whether individual patients who experienced complications received antibiotics. Conclusions might differ if individual level analyses showed that complications arise in patients who were treated with antibiotics. We did not evaluate the outcomes of individual patients identified as having complications in this study. Further research is required to evaluate the severity of complications, such as pneumonia, and their outcomes, including mortality. Future studies might also make use of linked hospital episode data, which in recent years have become available for selected CPRD practices in England to evaluate patients in more detail who have been admitted to hospital. The risk of complications associated with different classes of antibiotics also merits study. We acknowledge that there may be other complications, such as a proportion of all cases of septicaemia diagnosed in primary care, which might follow from an RTI. We acknowledge several sources of misclassification: we used a sample of the UK Clinical Practice Research Datalink (CPRD) to estimate consultation and prescribing rates; there is variation among general practices in the use of diagnostic categories^40^; general practice populations may vary in their use of out-of-hours and emergency services, whose generally higher antibiotic prescribing may not be captured in CPRD; and some general practices may use delayed antibiotic prescribing strategies,^4^ but these were not distinguished in the analysis of prescriptions issued. Use of near patient testing might possibly have contributed to better diagnosis during the period. These forms of misclassification generally tend to diminish estimated associations but might cause bias if effects are differentially distributed across prescribing categories. Diagnostic coding may have a subjective element^41^ and bias might arise if low prescribing practices are more likely than high prescribing practices to code pneumonia to justify the prescription of an antibiotic. The research utilised non-randomised data, and we adjusted for age, sex, region, deprivation category, and general practice, but it is possible unmeasured confounders might have biased the reported associations. We also caution that, in the analysis of large datasets “significant” results must be judged in relation to their clinical importance. Antibiotic prescribing in the UK is high compared with some international comparators, and we cannot be sure that the associations reported here would also hold at very low antibiotic prescribing levels.

### Meaning of the study: possible explanations and implications for clinicians and policymakers

This study provides evidence that general practices prescribing antibiotics less often at consultations for RTIs may experience a slight increase in the incidence of pneumonia and peritonsillar abscess, both of which would be expected to respond to treatment while bacterial pathogens remain sensitive to antibiotics. No increase is likely in mastoiditis, empyema, meningitis, intracranial abscess, or Lemierre’s syndrome. Even a large reduction in antibiotic prescribing was predicted to be associated with only a small increase in numbers of cases observed over a 10 year period, and this would be expected to reduce the risks of antibiotic resistance, the side effects of antibiotics, and the medicalisation of largely self limiting illnesses. The safety outcomes of no antibiotic prescribing strategies for RTIs are an important aspect for communication to patients and the public in the context of wider communication strategies to support antimicrobial stewardship.^42^

### Unanswered questions and future research

Further research is needed to quantify associations based on individual patient characteristics and consultation patterns in primary care, particularly in children and older adults, as we used age standardised prescribing measures. However, associations might vary in different age groups. We also recommend that future randomised studies should be sufficiently large to evaluate safety outcomes of strategies to reduce antibiotic prescribing.

**What is already known on this topic**

Widespread unnecessary utilisation of antibiotics is leading to an increase in antimicrobial drug resistance
Many respiratory tract infections (RTIs) are largely self limiting, but antibiotics continue to be prescribed for about 50% of consultations for RTIs in primary care
RTIs are infrequently associated with complications, including pneumonia, peritonsillar abscess, mastoiditis, meningitis, intracranial abscess, and Lemierre’s syndrome, but it is not known whether these are more common in general practices that prescribe antibiotics less often

**What this study adds**

General practices prescribing antibiotics less often for RTIs had slightly higher rates of pneumonia and peritonsillar abscess than higher prescribing practices
There was no evidence that mastoiditis, empyema, meningitis, intracranial abscess, or Lemierre’s syndrome were more frequent at low prescribing practices
Even a substantial reduction in antibiotic prescribing was predicted to be associated with only a small increase in numbers of cases observed, and this would be expected to reduce the risks of antibiotic resistance, the side effects of antibiotics, and the medicalisation of largely self limiting illnesses

## Notes

Ethical approval: The protocol for the study was approved by the Clinical Practice Research Datalink (CPRD) independent scientific advisory committee, reference 14_130A2. The CPRD has broad National Research Ethics Service Committee ethics approval for observational research studies. The research was conducted to inform a study funded by the NIHR Health Technology Assessment programme. The outcomes were selected from a wider range of safety outcomes proposed for the study because they were identified as being potentially associated with respiratory tract infections.

Data sharing: CPRD data were analysed under licence and are not available for sharing.

Transparency: The guarantor (MCG) affirms that the manuscript is an honest, accurate, and transparent account of the study being reported; that no important aspects of the study have been omitted; and that any discrepancies from the study as planned (and, if relevant, registered) have been explained.
